# Avian Influenza Risk Perception among Poultry Workers, Nigeria

**DOI:** 10.3201/eid1504.070159

**Published:** 2009-04

**Authors:** Folorunso O. Fasina, Shahn P.R. Bisschop, Ademola A. Ibironke, Clement A. Meseko

**Affiliations:** National Veterinary Research Institute, Vom, Nigeria (F.O. Fasina, C.A. Meseko); University of Pretoria, Pretoria, South Africa (F.O. Fasina, S.P.R. Bisschop); Osun State Ministry of Agriculture, Osun, Nigeria (A. A. Ibironke)

**Keywords:** Avian influenza, Nigerian farmers, poultry workers, human infection, letter

**To the Editor:** In Nigeria and other African countries, outbreaks caused by the Asian strain of highly pathogenic avian influenza virus (HPAI) subtype H5N1 have occurred in poultry. These countries do not have the capacity to effectively manage, eliminate, and control animal diseases, and humans generally live in close contact with poultry (*1**,**2*).

Before these outbreaks (2006) in other countries, effective risk communication had reduced chances of human infection (*3**,**4*), and the effect of news media reports in reducing infection also had been reported (*5*). However, risk evaluation, perception, or communication has not been reported from Africa, where poverty (*6*), inadequate primary healthcare facilities (*7**,**8*), and nonchalant attitudes to animal diseases predominate. In this study, we report the perception of poultry workers in Nigeria to avian influenza (AI).

To determine perception of AI, from November 2006 through January 2007, we surveyed a random sample of 200 poultry workers in 8 of the Nigerian HPAI virus (H5N1)–affected states: Kaduna and Kano (north); Plateau, Bauchi, Nasarawa, and Abuja (central); and Ogun and Lagos (south). We used pretested and previously evaluated structured interviews. Telephone interviews were used to confirm data collected from ≈15% of respondents, and data were evaluated by using descriptive statistics. All responses were evaluated according to published guidelines of the World Organisation for Animal Health (OIE), US Centers for Disease Control and Prevention, World Health Organization, OIE/Food and Agriculture Organization of the United Nations (FAO) Network on Avian Influenza, and Food and Drug Administration of the United Nations, taken from the organizations’ websites.

One hundred thirty-five (68%) poultry farmer workers from 36 infected and 39 uninfected flocks responded to the interview. Farms evaluated had flocks of a few hundred (200–300) to >70,000 chickens. Eighty-nine percent of respondents were concerned about AI; 57% knew that AI has food safety implications. Eighteen percent were willing to eat chicken that had died or gotten sick from infection; 21% would eat chicken and eggs from infected farms. These surveyed workers stated that thorough cooking, frying, cleaning, and traditional cooking methods were sufficient to kill the HPAI virus; 23% were not aware of risk associated with processing of HPAI-infected meat.

Although 61% reported knowing some risk factors for AI (e.g., close association with infected birds, home slaughtering, unprotected personnel, eating and processing of infected carcasses), only 56% correctly described some risk factors. Sixty percent reported having heard about the AI virus before the outbreak in Nigeria; 55% reported knowing the symptoms in affected birds. Of the 67% who had some knowledge of the symptoms, 56% were familiar with differential diagnoses. Ninety percent erroneously believed AI was fatal only to birds, although 58% believed it could affect humans.

After the first wave of HPAI outbreaks in poultry in Nigeria (2006), 98% of respondents said they had gained some knowledge about AI, primarily through television but also through radio, newspapers, government, community public health messages, veterinarians, and the Internet or through journals and seminars. Although 21% of respondents had had their flocks tested for AI, they had difficulty distinguishing between clinical assessment and laboratory tests. Seven farmers had themselves been tested for AI exposure. Seventy-six percent of farmers were willing to be tested, but only 67.9% were willing to have their flocks tested.

Respondents were more concerned about the effect of AI on financial preservation of business interests than on public health risk. Knowledge about biosecurity and risk factors varied widely between urban/periurban (51 correct answers) and rural (25 correct answers) workers. Most correct answers about knowledge of human infection by the HPAI virus also came from urban/periurban respondents. Forty percent of respondents who would not eat AI-infected chicken cited religious prohibition to eating dead animals. Seven respondents did not believe AI exists at all and viewed the outbreak situation as a diversionary tactics from the 2007 presidential election.

Our findings are similar to trends reported among poultry workers in previous studies (*3**,**4*) (Table). Our study showed that knowledge of food safety and risk factors and differentiation between HPAI and other poultry diseases is poor among the poultry farming communities of Nigeria. The belief by 90% of respondents that AI is lethal only in poultry further increases risk for human infection. The study also showed that farmers believe the news media (broadcast and print) are important in increasing public understanding of AI. Nearly all respondents agreed that poultry enterprise is profitable, albeit risky, and were not willing to abandon the business even in the event of an AI outbreak. Because the knowledge gap between the rural and urban communities further heightens the risk for human AI infection in Nigeria, public health messages about AI should target rural communities.

**Table:** Comparison of positive responses to avian Influenza questionnaire, November 2006–January 2007*

Item	Nigeria	Italy	Thailand
Surveyed population	Farmers/farm workers	Farm workers	Consumers
Food safety knowledge	56.8%	58%	92%
Avian influenza concern	88.6%	69.7%	6
Knowledge of avian influenza	67.1%	63.8%	88%
Literacy level of respondents	47.7% higher education (degree, tertiary education)	One third high school and college	98%
Receipt of information after outbreaks	97.5%	91.8%	NA
Sources of information	TV, radio, newspaper	Mass media/health Worker/employers	NA
Economics of poultry production	Unwilling to quit poultry production in event of outbreaks	NA	Very important economic source
Percentage of urban respondents	40.2%	NA	41.9%

*NA, not available.

Previously, workers have indicated that socioeconomic factors prevent the rural and urban poor from accessing healthcare facilities (*8*). Lack of access to healthcare was evident in the response of workers who stated they would want to have themselves and their flocks tested if healthcare services were available and if government agencies would bear the cost of tests that may be unaffordable to most.

Since this survey, progress in disseminating knowledge of AI in Nigeria has been substantial. The country has established desk offices (state centers for coordination of surveillance activities in animals) to carry out regular surveillance for HPAI virus (H5N1), and farmers have tremendously improved their knowledge (*9*).
